# Divergent regulation of rice yield by carbon nanomaterials via biomass allocation and seed setting rate

**DOI:** 10.3389/fpls.2026.1779312

**Published:** 2026-03-17

**Authors:** Yutong Ma, Hao Chen, Zhun Tian, Rui Wang, Zihan Sun, Tongxin Li, Ming Zhang, Yu Wang

**Affiliations:** 1 State Key Laboratory of Soil and Sustainable Agriculture, Institute of Soil Science, Chinese Academy of Sciences, Nanjing, China; 2 Shandong Agriculture University, Tai’an, China; 3 Nanjing University of Information Science and Technology, Nanjing, China; 4 Anhui Academy of Forestry, Hifei, Anhui, China; 5 University of Chinese Academy of Sciences, Beijing, China

**Keywords:** biomass allocation, carbon nitride, entire rice growth stages, graphene, reproductive stage, seed setting rate, vegetative stage

## Abstract

**Introduction:**

Nanomaterials (NMs) technology has shown great potential in sustainable agriculture. However, comprehensive assessments of their impacts on the entire rice growth stages, particularly from vegetative to reproductive periods, are still limited.

**Methods:**

Herein, two carbon NMs, graphitic carbon nitride (C_3_N_4_) and graphene, were applied via daily foliar spraying for 7 days at the rice tillering, jointing, flowering and ripening stages to systematically investigate their effects on rice growth dynamics.

**Results:**

Our results revealed that both C_3_N_4_ and graphene primarily affect rice growth during reproductive stages (flowering and ripening) rather than vegetative stages (tillering and jointing), with contrasting effects. C_3_N_4_ inhibited the transfer of dry biomass from vegetative organs (stems and leaves) to reproductive organs (panicle and grains), resulting in a marked reduction in seed setting rate by 34.5% and consequently a sharp yield decline by 45.1%. Conversely, graphene facilitated the allocation of more biomass to reproductive organs than vegetative organs, and increased seed setting rate by 10.7%, resulting in 11.8% higher rice yield.

**Discussion:**

Our findings underscore the differential influence of carbon NMs on rice growth mediated through modulation of biomass partitioning and seed setting rate, which is of significant relevance for developing nano-enabled strategies to promote global food security.

## Graphical Abstract

**Figure:**
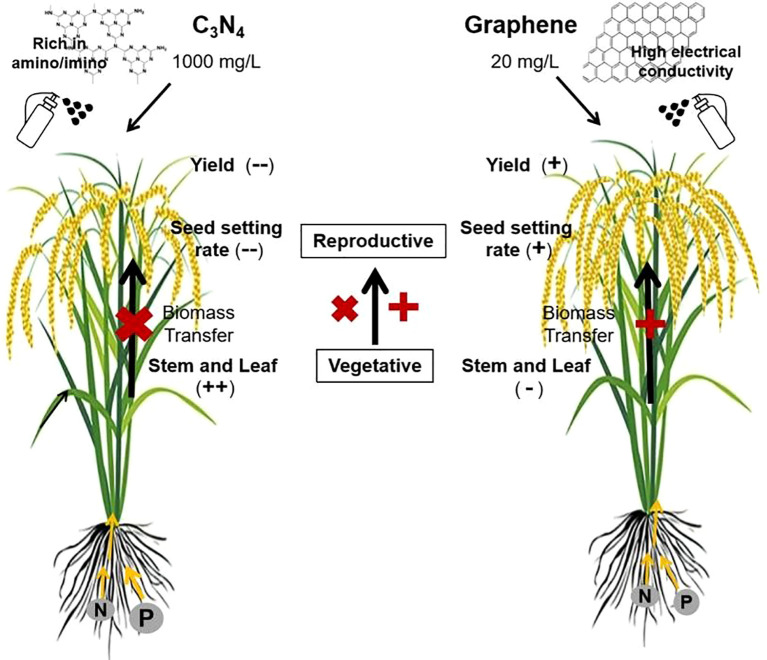

## 1 Introduction

In recent years, nanomaterials (NMs) have emerged as a revolutionary tool with substantial potential to enhance agricultural sustainability for ensuring global food security (Pulizzi, 2019). A meta-analysis indicated that nano-enabled agricultural practices can increase food plants production by 20.0%-30.0% (Gomez et al., 2021; Wang et al., 2020) compared to traditional fertilizers due to more efficient nutrient uptake, enhanced photosynthesis and better crop protection strategies (Jiao et al., 2023; Kumar et al., 2023). Rice (*Oryza sativa L.*), a primary source of calories for over half of the global population (Yuan et al., 2021; Chen et al., 2024; Zhang et al., 2024), has shown significant potential for yield enhancement with application of NMs (Cao et al., 2025; Guo et al., 2024; Chen et al., 2022).

Carbon NMs, such as carbon nitride (C_3_N_4_) and graphene, have gradually gained considerable attention due to their excellent photothermal conductivity and unique physicochemical properties (Chen et al., 2022; Yoo et al., 2023; Abd-Elsalam, 2020; Ismael, 2020). For example, Ma et al. (2021) demonstrated that C_3_N_4_ significantly increased rice shoot and root fresh weight by 22.4%-29.9% by enhancing nutrient assimilation and regulating plant nutrient metabolism. Graphene not only facilitates nutrient uptake in maize, but also up-regulates genes related to nitrogen and potassium metabolism, as well as phytohormone signaling (Zhang et al., 2025). Additionally, Lu et al. (2020) reported that graphene improves electron transfer and reactive oxygen species (ROS) scavenging in Photosystem II (PSII), significantly enhancing photo-phosphorylation efficiency in rice (Suganami et al., 2021). However, most current studies primarily focus on the impact of NMs on a single rice growth stage, lacking a comprehensive assessment of the whole growth stages, particularly the transition from vegetative to reproductive stages, which are heavily influenced by light and thermal conditions.

The whole growth period of rice includes vegetative growth and reproductive growth (Liang et al., 2014; Xing et al., 2025). Photosynthetic products serve as the foundation for rice growth and dry matter formation, with rice yield being determined by both dry matter transport during the vegetative growth stage and the accumulation of assimilation products during the reproductive stage (Wen et al., 2024; Xu et al., 2024). Pre-flowering dry matter translocation primarily contributes to the formation of rice structural materials, while post-flowering assimilation product accumulation is directly linked to yield formation, accounting for 60.0% to 80.0% of grain yield (Fageria, 2004; Wu et al., 2018). Moreover, soil nutrients—particularly carbon (C), nitrogen (N), and phosphorus (P)—play vital roles in rice growth, with distinct requirements during the vegetative and reproductive phases. C is the primary building block for photosynthesis and plant dry matter, affecting early rice growth and plant health (Yoshida et al., 2023). N primarily drives vegetative growth by enhancing leaf expansion and chlorophyll synthesis, which boosts photosynthetic capacity; however, excessive N during the reproductive phase may shift biomass allocation towards foliage at the expense of grain development (Evans and Clarke, 2019; Mu and Chen, 2021). P is crucial for jointing and stress resilience, with its availability during the reproductive phase directly influencing panicle size and seed quality (Lambers, 2022). It is noteworthy that most foliar-applied NM studies focus on crop physiological responses and leaf processes, often overlooking the complex interplay between nanomaterials, fertilizers and soil nutrients dynamics. Rice yield formation depends not only on carbon assimilation but also on the timing and efficiency of assimilate and nutrient partitioning between vegetative organs and reproductive organs. The transition from vegetative growth to reproductive growth is a developmental bottleneck during which source capacity, nutrient acquisition, and sink establishment must be tightly coordinated. Consequently, evaluating NMs effects across multiple growth stages—while simultaneously tracking biomass allocation and seed-setting rate—is essential to distinguish “photosynthesis-enhancing” effects from true yield formation mechanisms.

Herein, C_3_N_4_ and graphene were applied as daily foliar sprays for 7 consecutive days at the tillering, jointing, flowering and ripening stages of rice, to investigate their impacts on photosynthetic characteristics, nutrient uptake, dry matter accumulation and allocation, as well as grain yield. This multi-stage design enables a direct comparison of whether NMs primarily enhance source capacity, strengthen reproductive sink development, or modify source–sink coordination across the rice life cycle. We tested three hypotheses: (1) both C_3_N_4_ and graphene enhance leaf photosynthetic performance at key developmental stages, (2) C_3_N_4_ preferentially increases vegetative biomass and nutrient retention, shifting dry matter partitioning toward stems and leaves and thereby limiting reproductive allocation and yield gains; and (3) graphene improves source–sink coordination by promoting nutrient uptake and assimilate translocation to reproductive organs, resulting in higher seed-setting rate and improved yield. These findings are expected to deepen the understanding of the stage-specific effects of carbon NMs on rice growth and development, and to provide valuable insights for developing sustainable nano-enabled strategies to enhance global food security.

## 2 Materials and methods

### 2.1 Materials

C_3_N_4_-based nanomaterials were synthesized via thermal polymerization. Briefly, 2 g urea was dissolved in 10 mL ethanol with 12 mg copper (II) disodium ethylenediaminetetraacetate tetrahydrate, stirred at 60°C for 4 h, dried, and heated to 550°C for 4 h to obtain Cu-CN. The control CN was prepared using the same procedure without copper salt (Hao et al., 2023). Biomass-derived graphene was fabricated by flash joule heating. Rice straw, maize straw, sawdust, and bamboo powder were pyrolyzed at 750°C for 120 min under N_2_ flow to produce biochar. Then 0.2 g biochar was compressed and treated with alternating current and direct current under vacuum to yield graphene (Jiao et al., 2025).

### 2.2 Greenhouse experiment

The pot experiment was conducted from July to December 2023 at the Agro-meteorological Experimental Station of Nanjing University of Information Science and Technology (NUIST). The experimental soil was collected from a paddy field at the National Agroecosystem Observation and Research Station, Chinese Academy of Sciences, Changshu (31°16’N, 119°54’E). The rice cultivar test was *Oryza sativa* L. cv. Nanjing 46. Soil analysis revealed the following properties: pH 5.4 (soil: water = 1:2.5), total carbon (TC) 9.58 g kg^-1^, total nitrogen (TN) 1.01 g kg^-1^, total phosphorus (TP) 0.73 g kg^-1^, dissolved organic carbon (DOC) 74.7 mg kg^-1^, organic nitrogen (DN) 10.7 mg kg^-1^, and available phosphorus (AP) 57.9 mg kg^-1^.

A field pot experiment was conducted with three experimental treatments, each implemented across four growth stages and arranged with three replications per stage, resulting in a total of 36 pots. The three treatments were set as follows: conventional fertilization (CK), foliar application of C_3_N_4_ and graphene. The concentrations of C_3_N_4_ (1000 mg·L^-1^) and graphene (20 mg·L^-1^) used in this study are the most effective concentrations in our previous study (Hao et al., 2023; Jiao et al., 2025). Prior to pot filling, the collected soil samples were air-dried, then mechanically ground and passed through a 2-mm mesh sieve for homogenization. Each pot was filled with 2 kg of the prepared soil, and all potted plants were irrigated with 200 mL of water every two weeks to maintain consistent soil moisture conditions.

Prior to rice transplantation, equivalent quantities of N, P, and potassium fertilizers were uniformly applied to all treatments. The experimental foliar fertilizers were prepared by subjecting the NMs to ultrasonic treatment for 10 minutes after dispersion in purified water, followed by application using a handheld plastic spray bottle. The fertilizers employed in this investigation consisted of urea, calcium superphosphate, and potassium chloride. The application rates of N, phosphorus (as P_2_O_5_), and potassium (as K_2_O) for each cropping season were 240 kg ha^-1^, 60 kg ha^-1^, and 60 kg ha^-1^.

The foliar sprays containing C_3_N_4_ nanoparticles and graphene were applied until near-complete leaf surface wetting was achieved, each treatment application was performed once during each growth period, the CK treatment received no foliar fertilizer application. To mitigate the effects of solar radiation and elevated temperatures on foliar absorption of NMs, spraying operations were conducted during predawn hours, synchronized with sunrise, during precipitation events, the potted plants were transferred to a protected environment. Rice transplantation was performed in July using two seedlings per pot (with two planting holes per pot), and harvesting was completed in October utilizing destructive sampling methodology.

### 2.3 Gas exchange and photosynthesis parameters

Measurements were conducted between 9:00 a.m. and 11:30 a.m. on days with optimal sunlight conditions. Flag leaves were sampled from different pots at four growth stages: tillering, jointing, flowering, and ripening stages. Photosynthetic measurements and biomass sampling were performed on distinct rice plants. Specifically, three rice plants were randomly selected from the replicated pots of each treatment for the determination of photosynthetic parameters, and another three synchronously growing plants were chosen for biomass sampling. This experimental design avoided the adverse effects of leaf damage caused by photosynthetic measurements on subsequent biomass determination of the same plant, thus ensuring the independence and accuracy of the two sets of indicators. Photosynthetic parameters were measured using a portable photosynthesis system (LI-6800, LI-COR, Lincoln, NE, USA). The photosynthetically active radiation (PPFD) was maintained at 1,500 μmol m^-2^ s^-1^, with the CO_2_ concentration set at 410 μmol mol^-1^. After achieving stable reading, the following parameters were recorded: net photosynthetic rate (*P_N_*), stomatal conductance (*gs*), transpiration rate (*E*), intercellular carbon dioxide concentration (*C_i_*), and transpiration efficiency (*E rate*).

For chlorophyll analysis, leaf discs (0.7 cm diameter) were excised using a cork borer from leaves selected for photosynthetic measurements. The samples were immersed in 2 mL of 95% ethanol and stored in darkness at 4°C until complete pigment extraction was achieved. Absorbance measurements were performed at 664 nm, 649 nm, and 470 nm using a spectrophotometer. Chlorophyll a (chl a) concentration was calculated using Equation 1, and chlorophyll b (chl b) concentration was determined via Equation 2. Total chlorophyll (chl) concentration was computed according to Equation 3, while carotenoids (car) concentration was calculated using Equation 4.

(1) $$\begin{array}{c} \text{Chl}\;\text{a}\;\left(\text{mg}\;{\text{m}}^{-2}\right)\;=13.95{\text{A}}_{665}-6.80{\text{A}}_{649} \end{array}$$

(2) $$\begin{array}{c} \text{Chl}\;\text{b}\;\text{mg}\;{\text{m}}^{-2}=24.96{\text{A}}_{649}-7.32{\text{A}}_{665} \end{array}$$

(3) $$\begin{array}{c} \text{Chl}\;\text{mg}\;{\text{m}}^{-2}=18.16{\text{A}}_{649}+6.63{\text{A}}_{665} \end{array}$$

(4) $$\begin{array}{c} \text{Car}\;\left(\text{mg}\;{\text{m}}^{-2}\right)=\frac{1000.00{\text{A}}_{470}-2.05\text{Chl}\;\text{a}-114.80\text{Chl}\;\text{b}}{248.00} \end{array}$$

### 2.4 Rice nutrient growth parameters

Plant height was measured at four key growth stages, namely the tillering, jointing, flowering and ripening stages, with a standardized measuring ruler following a strict operational protocol. Specifically, plant height was defined as the vertical distance from the soil surface to the tip of the flag leaf to eliminate errors caused by inconsistent measuring positions. All height measurements were conducted by the same investigator and uniformly carried out between 8:00 and 10:00 a.m. to avoid minor fluctuations in plant height induced by diurnal transpiration-induced water loss in rice plants. The collected plant samples were thoroughly rinsed with deionized water to remove surface impurities, after which the stems and leaves were separated for individual morphological and physiological analyses (Liu et al., 2023).

For dry weight determination, plant samples underwent a standardized drying protocol: initial pre-drying at 65°C for 12 hours, followed by a killing phase at 105°C for 30 minutes, and subsequent continuous drying at 65°C until constant weight was achieved. The stabilized dry weights were then recorded. All dried plant samples were homogenized to fine powder using a grinder and ball mill for subsequent nutrient analysis. TC and TN contents were determined by dry combustion method using a Vario MAX CNS elemental analyzer (vario MACRO CN, Elementar Analysensysteme GmbH, Germany). TP content was measured following sulfuric acid-perchloric acid digestion, with quantification performed using a fully automated Smartchem analyzer (Smartchem 200, AMS/Westco, Italy).

### 2.5 Soil nutrient indicators

Soil samples were collected from three randomly selected pots per treatment at four growth stages: tillering, jointing, flowering, and ripening stages of rice. Subsequently, soil samples from different growth periods were processed by removing fresh soil impurities. The soil samples were then air-dried at room temperature and mechanically ground for subsequent chemical analyses.

The TC and TN contents of the soil samples were determined using the dry combustion method with a vario MAX CNS elemental analyzer (vario MACRO CN, Elementar Analysensysteme GmbH, Germany). The TP content was measured following concentrated sulfuric acid-hydrogen peroxide digestion and subsequent ultraviolet-visible spectrophotometric analysis (UVmini-1240, Shimadzu Corporation, Japan).

DOC and dissolved DN concentrations were quantified using a total organic carbon analyzer (multi-N/C 3100, Analytik Jena AG, Germany). Soil available phosphorus (Olsen-P) was extracted with 0.5 M sodium bicarbonate solution (pH 8.5), while TP was determined after ashing and 0.5 M sulfuric acid treatment, both measured using an ultraviolet-visible spectrophotometer (UVmini-1240, Shimadzu Corporation, Japan).

### 2.6 Rice yields

Rice grains were sampled during the late reproductive stage, and the number of panicles per pot were recorded at the time of sampling. The grains were subsequently manually threshed and separated into two distinct categories: filled grains and unfilled grains. Both grain types were individually weighed and counted.

### 2.7 Data analysis

The data were collected and analyzed using Microsoft Excel 2019 and IBM SPSS Statistics 22 software (SPSS Inc., USA). The figures were created using SigmaPlot software (version 12.0, MMIV, Systat Software, San Jose, CA, USA). Image plotting was performed using Origin 2023. The significance of differences in plant and soil physicochemical properties was assessed using an independent samples t-test in SPSS Statistics 22 (SPSS Inc., USA).

## 3 Results

### 3.1 Effects of carbon NMs on rice growth and photosynthetic characteristics

**Figure 1:**
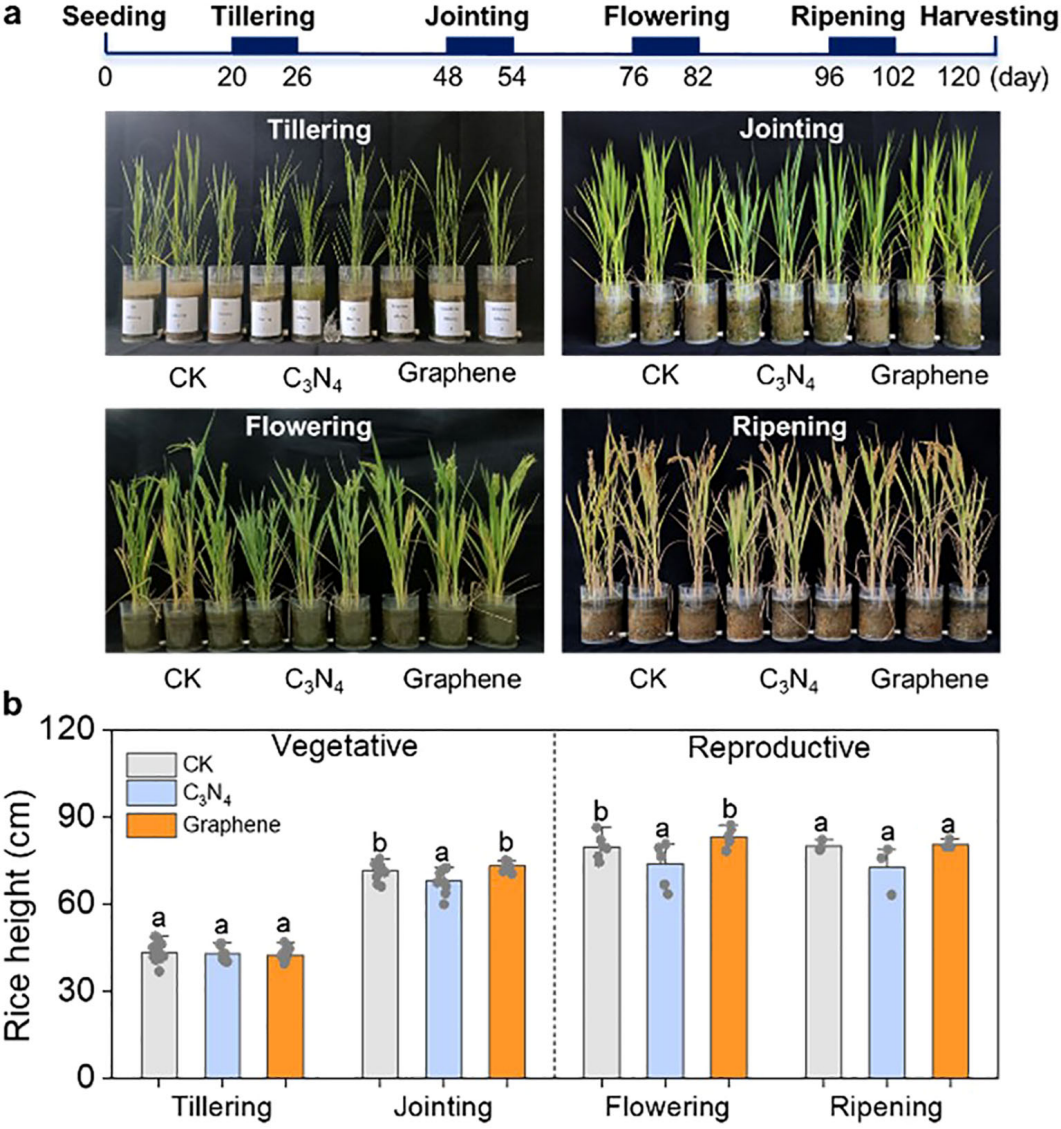
Schematic of the design and rice growth of the whole growth stages experiment. Phenotype of the whole rice growth stages **(A)** rice height at different stages under two carbon NMs **(B)**. Bars indicate the mean values ± standard deviation (SD). The lowercase letters above the bars indicate statistical significance of differences among the CK, C3N4, and graphene treatments. This labeling convention is uniformly applied for all measured parameters across rice growth stages in the figures.

To comprehensively assess the potential of carbon NMs in enhancing rice growth, rice plants at the tillering, jointing, flowering, and ripening stages were subjected to daily foliar application of C_3_N_4_ and graphene for 7 d (**Figure 1A**). Compared to CK, neither C_3_N_4_ nor graphene significantly affected rice height throughout the entire rice growth stages (**Figure 1B**).

**Figure 2:**
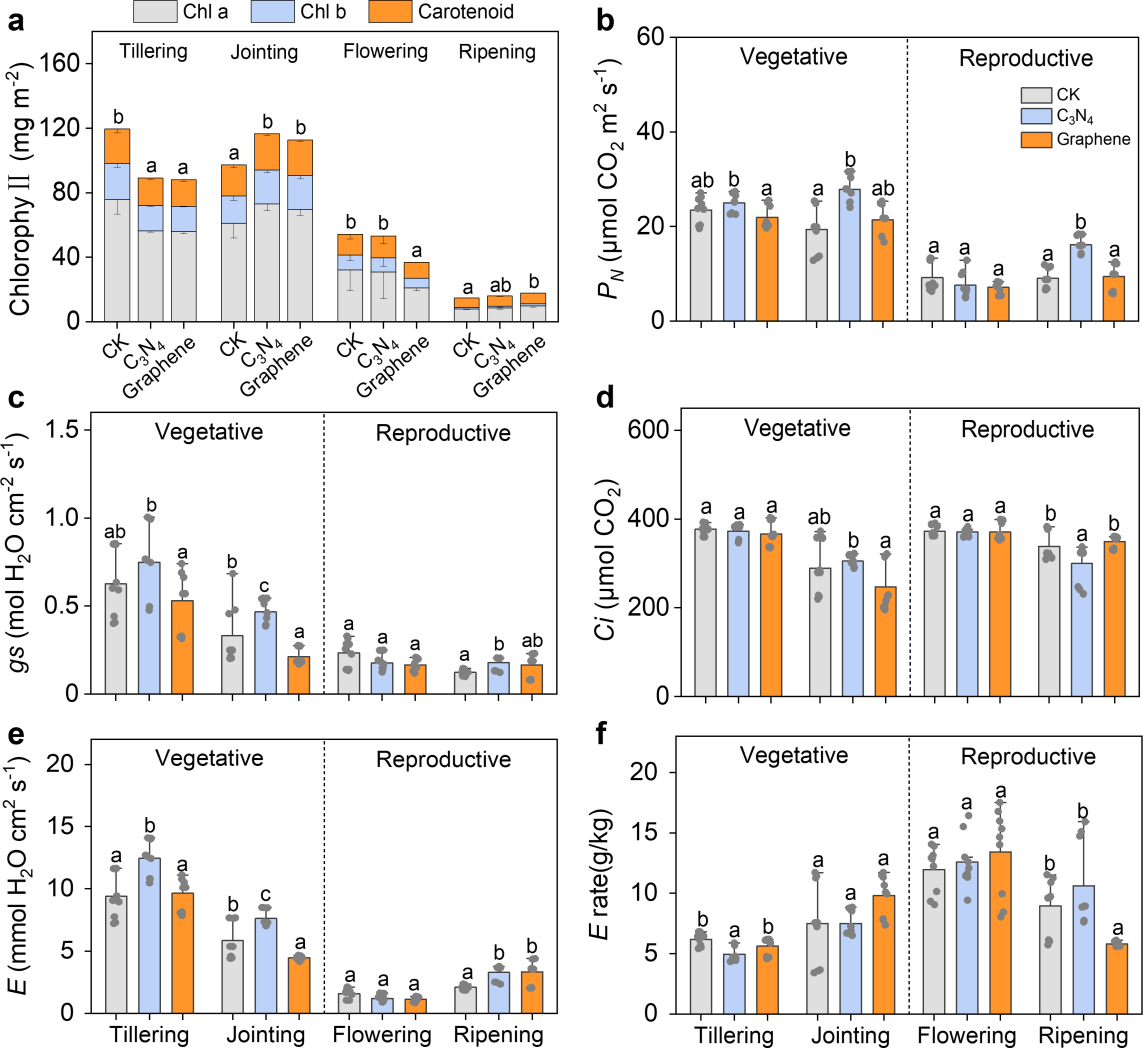
Changes of photosynthesis during the whole rice growth stages under two carbon NMs. The chlorophyll **(A)** net photosynthetic rate (*P_N_*) **(B)** stomatal conductance (*gs*) **(C)** the transpiration efficiency (*E*) **(D)** intercellular carbon dioxide concentration (*C_i_*) **(E)** transpiration efficiency (*E* rate) **(F)**. Bars indicate the mean values ± standard deviation (SD). The lowercase letters above the bars indicate statistical significance of differences among the CK, C3N4, and graphene treatments. This labeling convention is uniformly applied for all measured parameters across rice growth stages in the figures.

Both C_3_N_4_ and graphene notably affected Chlorophyll primarily during the vegetative stage rather than reproductive stage, while exhibiting contrary effects at tillering and jointing stages (**Figure 2A**). Furthermore, both NMs modulated key photosynthetic parameters, including *P_N_*, *gs*, *E*, and *C_i_*. Compared with CK, C_3_N_4_ markedly increased *P_N_*, *gs* and *E* by 43.7%, 77.9% and 19.6%, 45.0% and 9.7%, 54.5% at the jointing and ripening stages, respectively (**Figures 2B, C, E**). Graphene significantly decreased *gs* and *E* by 56.4% and 31.2% at jointing stage, while increased by 35.0% and 58.9% at ripening stage. Both NMs had no significant effect on *C_i_* (**Figure 2D**).

### 3.2 Effects of carbon NMs on rice nutrient uptake and soil nutrient supply

**Figure 3:**
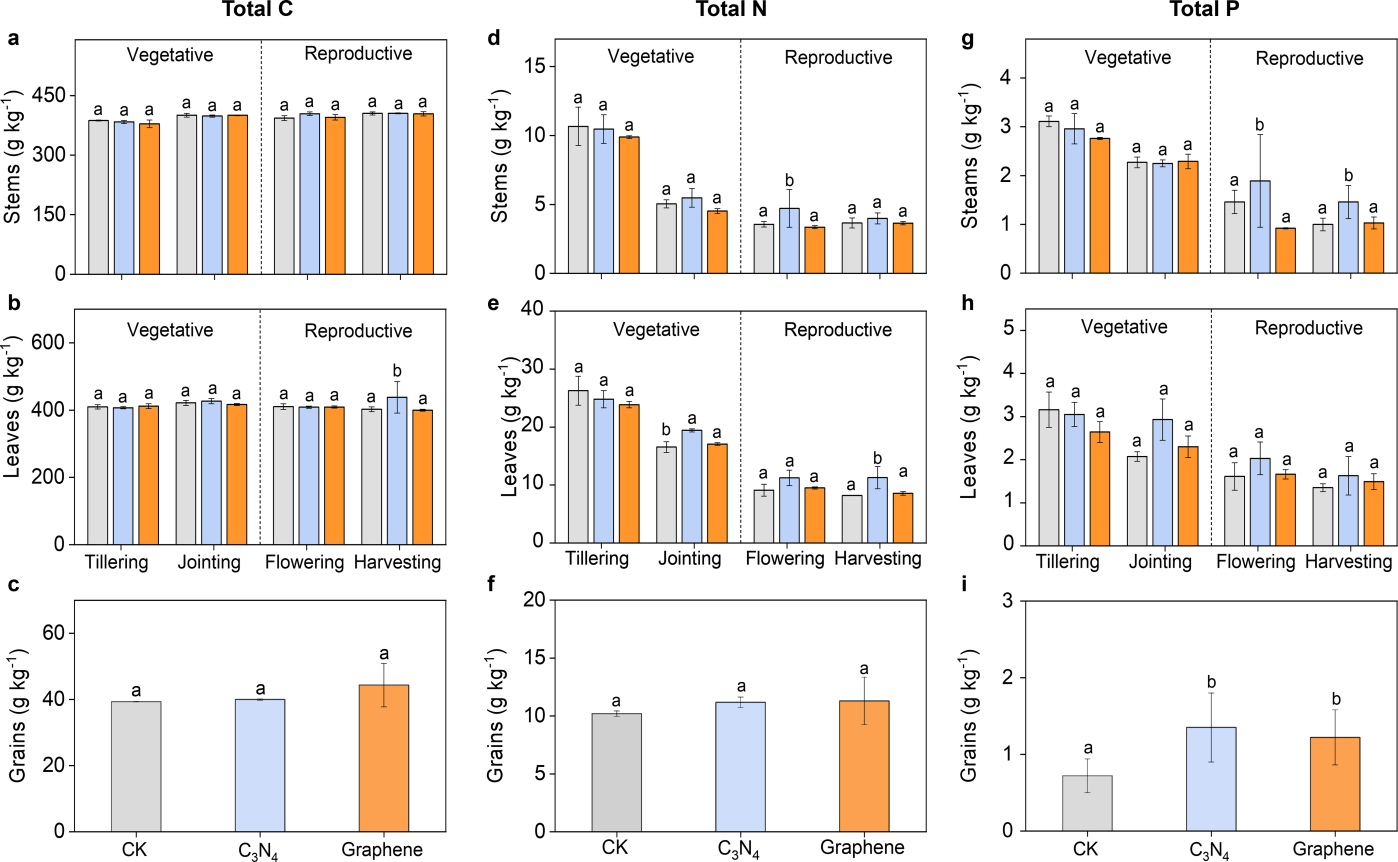
Effects of carbon NMs on rice nutrient uptake during the whole growth stages. Rice stems total C **(A)** Leaves total C **(B)** Grains total **(C)** Stems total N **(D)** Leaves total N **(E)** Grains total N **(F)** Stems total P **(G)** Leaves total P **(H)** Grains total P **(I)**. Bars indicate the mean values ± standard deviation (SD). The lowercase letters above the bars indicate statistical significance of differences among the CK, C3N4, and graphene treatments. This labeling convention is uniformly applied for all measured parameters across rice growth stages in the figures.

**Figure 4:**
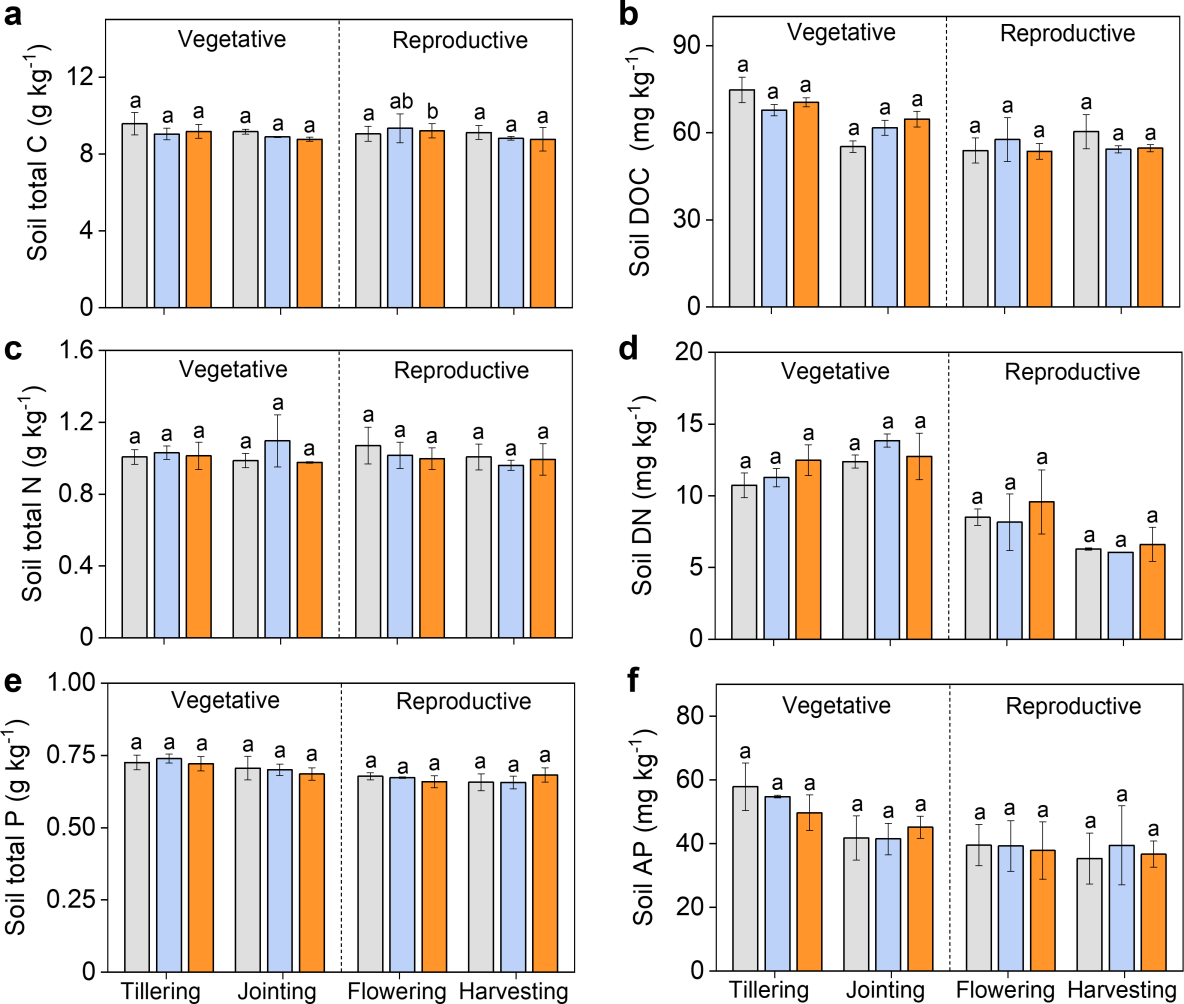
Changes of soil nutrient elements during the whole rice growth stages under two carbon NMs. Soil total C **(A)** Soil DOC **(B)** Soil total N **(C)** Soil DN **(D)** Soil total P **(E)** Soil AP **(F)**. Bars indicate the mean values ± standard deviation (SD). The lowercase letters above the bars indicate statistical significance of differences among the CK, C3N4, and graphene treatments. This labeling convention is uniformly applied for all measured parameters across rice growth stages in the figures.

Compared to CK, neither C_3_N_4_ nor graphene had significant effects on total C concentration of stems, leaves and grains throughout the entire rice growth stages (**Figures 3A-C**). However, C_3_N_4_ significantly increased total N of leaves and grains, and total P of stems and grains during reproductive period, indicating C_3_N_4_ facilitated N and P uptake. Graphene had no significant effect on N and P uptake in all organs throughout entire rice growth stages (**Figures 3D-I**). Moreover, both carbon NMs generally had little impact on soil total C, N and P content, and soil dissolved organic C and N, and available P except that dissolved organic N of crops significantly increased by 11.8% and 17.3% relative to CK at the jointing stage after C_3_N_4_ and graphene application (**Figure 4**).

### 3.3 Carbon NMs significantly changed dry biomass allocation

**Figure 5:**
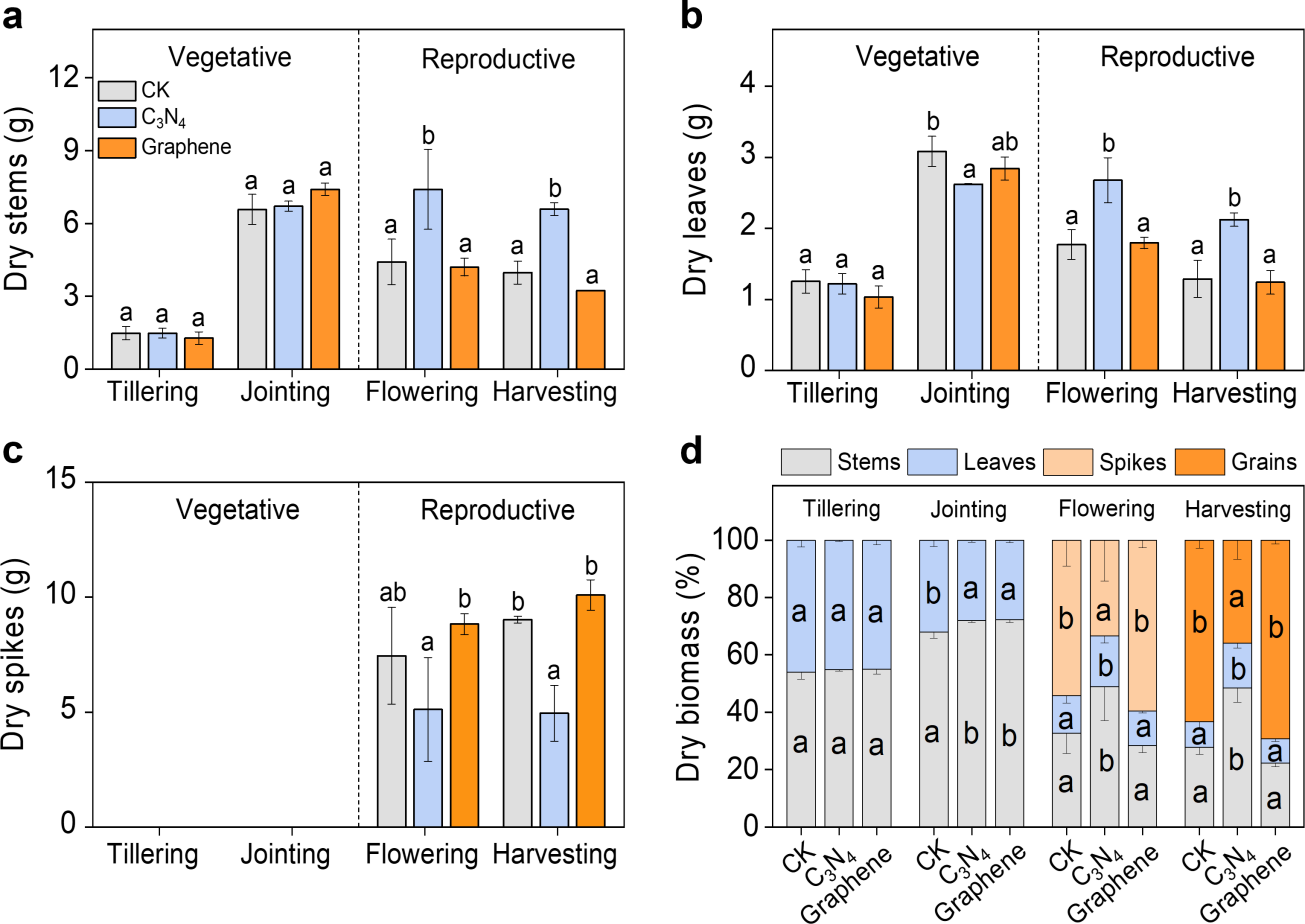
Carbon NMs significantly changed dry biomass allocation. Stem dry biomass during the whole rice growth stages (as below) **(A)** Leaves dry biomass **(B)** Panicle dry biomass **(C)** The percentage of stems, leaves, panicles and grains dry biomass **(D)**. Bars indicate the mean values ± standard deviation (SD). The lowercase letters above the bars indicate statistical significance of differences among the CK, C3N4, and graphene treatments. This labeling convention is uniformly applied for all measured parameters across rice growth stages in the figures.

Carbon NMs had little impact on the total dry biomass of rice plant, while significantly altered the internal allocation of dry biomass among different organs, especially from vegetative organs to reproductive organs. Compared to CK, C_3_N_4_ markedly increased dry biomass of stems and leaves by 67.7% and 51.2%, and 65.7% and 64.6% at flowering and harvesting stages, respectively (**Figures 5A, B**), while significantly decreased dry biomass of panicle by 45.1% at harvesting stage (**Figure 5C**). Graphene had little impact on the dry biomass of stems and leaves, but significantly increased panicle dry biomass (**Figures 5A-C**).

Compared to CK, C_3_N_4_ markedly increased the allocation of dry biomass to stems at jointing, flowering and harvesting stages, and markedly decreased the allocation of dry biomass to panicle at flowering stage, and to grains at harvesting stages. However, graphene exhibited contrasting results, significantly decreased the allocation of dry biomass to stems, and increased grains biomass at harvesting stages (**Figure 5D**). The results indicated that C_3_N_4_ inhibited the internal allocation of dry biomass from vegetative organs to reproductive organs, while graphene facilitated the internal allocation of dry biomass from vegetative organs to reproductive organs.

### 3.4 Carbon NMs affect rice yield by changing seed setting rate

**Figure 6:**
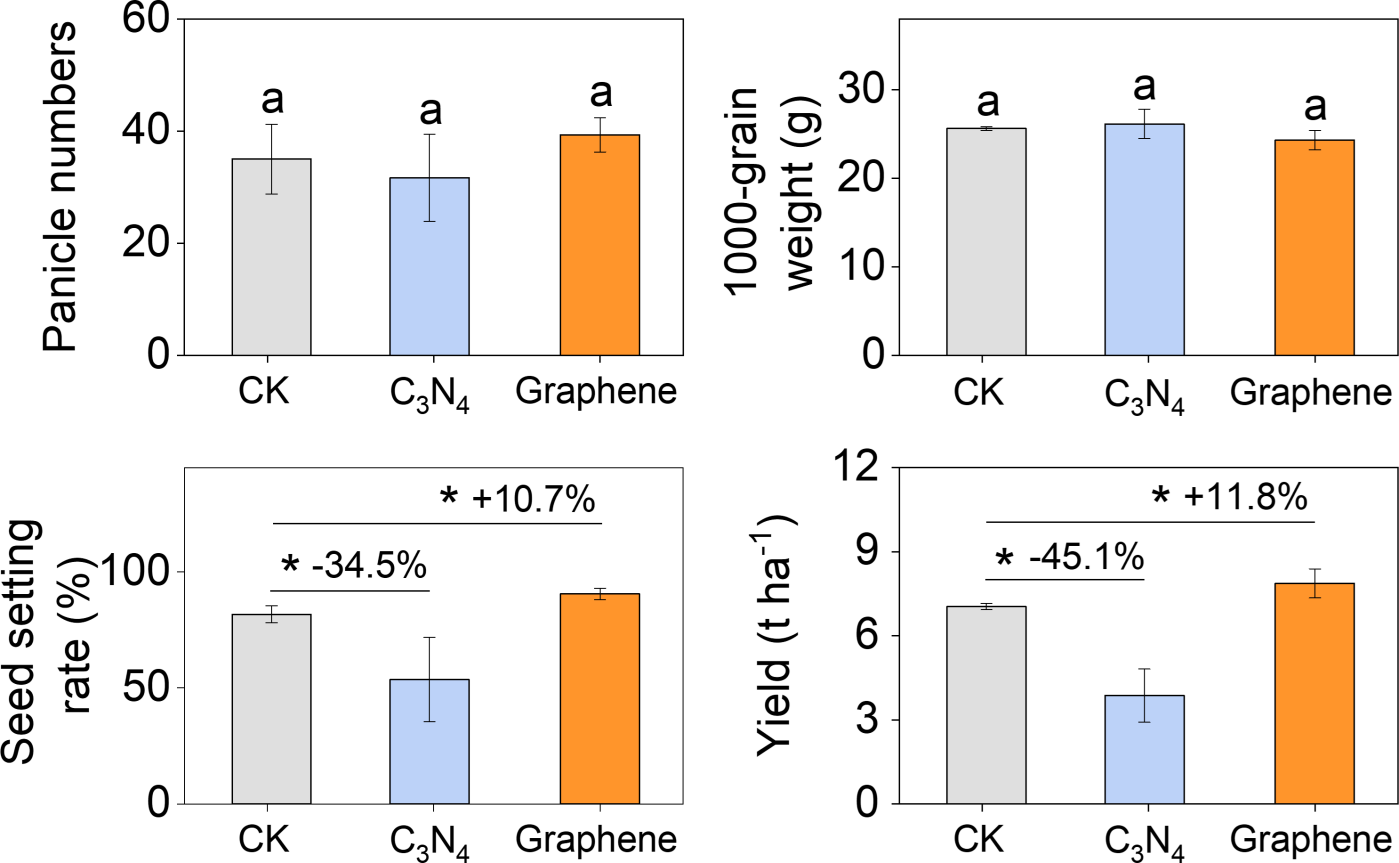
Carbon NMs affect rice yield by changing seed setting rate. Rice panicle numbers **(A)**; 1000-grain weight **(B)**; Seed setting rate **(C)**; Grain yield **(D)**. Bars indicate the mean values ± standard deviation (SD).

Rice yield mainly depends on the panicle numbers, thousand-grain weight, and seed setting rate. Our results showed that neither C_3_N_4_ nor graphene significantly affected the panicle numbers and thousand-grain weight (**Figure 6**), suggesting that neither C_3_N_4_ nor graphene adversely affected rice tillering. Interestingly, compared to CK, C_3_N_4_ showed a 34.5% lower seed setting rate but graphene showed a 10.7% higher rate (**Figure 6**), resulting in yields significantly lower by 45.1% and higher by 11.8%, respectively (**Figure 6**). The results indicated that C_3_N_4_ reduced rice yield mainly by decreasing seed setting rate due to suppressed reproductive growth, while graphene significantly facilitated rice reproductive growth, thereby boosting seed setting rate and enhancing grain filling.

## 4 Discussion

The enhanced photosynthetic performance observed after C_3_N_4_ application (**Figure 2**) may be explained by the interaction of C_3_N_4_-derived photogenerated electrons with plant cells at extracellular interfaces, which could shorten the effective electron-transfer route (Hao et al., 2024). Accordingly, we speculate—based on published evidence—that increased NADPH and ATP production provides additional energy for the Calvin–Benson–Bassham (CBB) cycle, thereby promoting CO_2_ fixation and the biosynthesis of photosynthetic pigments (Xue et al., 2024; Wang et al., 2023; Xiong and Flexas, 2020). Previous studies further suggest that the layered structure of C_3_N_4_ can function as an artificial antenna by emitting blue fluorescence that overlaps with the chloroplast absorption spectrum (Zhang et al., 2024; Jo et al., 2019). Consistently, Zhao et al. (2024) and Li et al. (2021, 2024) reported that this property enables C_3_N_4_ to harvest ultraviolet/blue light, facilitate electron transfer in PSII, shorten the electron-transport pathway, and expand the light-harvesting cross-section. Collectively, these effects may enhance *P_N_* and Rubisco activity, increase photosynthetic pigment content and leaf photochemical efficiency, and improve electron transport (Cao et al., 2018; Miao et al., 2023). In line with our results, C_3_N_4_ therefore appears to improve light-use efficiency and provide additional energy supporting rice growth, ultimately favoring the accumulation of photosynthetic products (Mathan et al., 2021; Yan et al., 2021). Compared to CK, C_3_N_4_ markedly reshaped the temporal distribution of nutrients between stems and leaves (**Figure 3**). Stem TC continued to increase even after panicle initiation, indicating sustained nutrient accumulation during the reproductive period (Wen et al., 2024). Moreover, C_3_N_4_ significantly altered TC/TP/TN content in both stems and leaves, underscoring its association with improved photosynthetic performance and nutrient acquisition (Huang et al., 2016). By contrast, graphene significantly enhanced N and P uptake by stems, leaves, and grains at the jointing and flowering stages (Xu et al., 2023). Because jointing represents the transition from vegetative to reproductive growth, we further speculate that the flowering stage benefits from improved nutrient partitioning under graphene application (Zhao et al., 2025).

Xu et al. (2024) showed that C_3_N_4_ can substantially alter assimilate partitioning, driving excessive allocation to vegetative organs at the expense of reproductive organs and ultimately reducing yield; this pattern is consistent with our results (**Figure 5**). In contrast, under graphene treatment, resources were preferentially allocated to reproductive growth and delivered to reproductive organs. This divergent response may be related to gradual N release mediated by amino (-NH_2_) and imino (-NH-) groups on the C_3_N_4_ surface, which could lead to excessive N supply during reproductive development and disrupt the balance of carbon and nitrogen metabolism in rice (Xu et al., 2024; Xue et al., 2023; Yang et al., 2025). Nitrogen is essential for chlorophyll biosynthesis and photosynthetic capacity (Yang et al., 2025). However, seed formation and grain filling during reproductive growth require substantial carbohydrate reserves (Blumstein et al., 2022). Excess N availability may redirect photosynthates toward protein and amino acid synthesis, thereby constraining seed development and grain filling (Aguirre et al., 2018). In our study, graphene improved nutrient uptake and utilization efficiency while enhancing photosynthesis and water-use efficiency, resulting in greater dry matter accumulation. Notably, the comparatively moderate influence of graphene on N metabolism coincided with increased grain dry matter accumulation, and we speculate that graphene may facilitate post-flowering translocation of assimilates from vegetative tissues to seeds, thereby increasing grain weight (Thorburn et al., 2024).

The contrasting biomass allocation patterns between the C_3_N_4_ and graphene treatments indicate a substantial shift in source–sink relationships in rice. Under C_3_N_4_, the increases in stem and leaf biomass at flowering (67.7%) and harvest (65.7%) point to a strong bias in carbon partitioning toward vegetative growth even during reproductive development (**Figure 5B**). This apparent disruption of the normal transition from vegetative to reproductive dominance suggests that C_3_N_4_ may interfere with hormonal signaling networks underlying this developmental switch (Takashi, 2001). The concomitant reduction in allocation to panicles and ultimately grains implies that C_3_N_4_ maintains a vegetative-priority state during reproductive stages, thereby compromising yield formation. Conversely, graphene promoted the transfer of assimilates from vegetative to reproductive organs, suggesting a fundamentally different interaction with developmental regulation (Sun et al., 2022; Zhou et al., 2020). Under graphene treatment, the preserved or enhanced development of reproductive organs indicates that the typical source–sink transition during flowering and grain filling is maintained or strengthened (Yu et al., 2015). The 10.7% increase in seed-setting rate under graphene (**Figure 6C**), a key indicator of successful pollination, fertilization, and early grain development, further supports improved reproductive performance. Consequently, the 11.8% yield increase (**Figure 6D**) suggests that graphene acts in a manner that aligns with—and reinforces—the plant’s developmental progression toward reproductive growth and grain filling (Gai et al., 2019).

Based on our physiological, allocation, and yield responses (**Figures 2**-**6**), we propose a working model in which C_3_N_4_ and graphene improve photosynthetic processes via distinct pathways but diverge sharply in their downstream consequences for source–sink coordination and yield formation. C_3_N_4_ appears to primarily act as a “source-enhancing” stimulus: its light-harvesting/photoredox features are consistent with improved photochemistry and carbon assimilation, which would increase assimilate availability during vegetative growth. C_3_N_4_ likely strengthens vegetative sinks (stems/leaves) or delays the establishment of reproductive sink dominance, thereby limiting assimilate remobilization to panicles and grains and ultimately constraining yield formation. In contrast, graphene is better described as a “source–sink co-optimizer.” While graphene also improves resource acquisition and photosynthetic performance, the key difference is that nutrient and dry matter allocation patterns indicate preserved or enhanced transfer from vegetative organs to reproductive organs during flowering and grain filling. The observed improvements in seed setting and yield components are therefore consistent with stronger reproductive sink strength and/or more efficient post-flowering assimilate translocation. Collectively, our findings highlight a potentially important principle for nano-enabled crop improvement: increasing photosynthetic capacity alone may be insufficient to raise yield unless sink development and assimilate partitioning are simultaneously supported. This framework provides a testable basis for future work, such as quantifying phloem transport capacity, carbon remobilization kinetics, hormone-related transition signals, and nitrogen assimilation dynamics to pinpoint the regulatory nodes through which C_3_N_4_ and graphene differentially reshape the rice source–sink network.

## 5 Conclusion

In this study, we used C_3_N_4_ and graphene to probe how carbon-based nanomaterials regulate source–sink relationships across the rice life cycle. The contrasting outcomes highlight that yield responses are not determined by enhanced carbon assimilation per se, but by whether assimilates and nutrients are effectively mobilized from vegetative tissues to the developing grain. In this framework, C_3_N_4_ primarily favors vegetative growth and nutrient retention, which can weaken reproductive allocation and ultimately constrain yield formation, whereas graphene tends to support reproductive sink strength and/or assimilate translocation, improving the efficiency with which pre-anthesis resources are converted into grain production.

These findings carry two broader implications. First, carbon nanomaterials should be evaluated and optimized as stage-dependent regulators of whole-plant carbon partitioning rather than as universal “growth promoters,” with reproductive performance serving as a key endpoint. Second, the C_3_N_4_-driven shift toward vegetative biomass suggests clear value beyond grain systems: it may be better positioned for biomass-oriented applications and for crops where economic yield derives mainly from stems and leaves (e.g., sugarcane, tea, tobacco, and leafy vegetables). Future work should clarify the mechanistic basis of these divergent source–sink effects and define crop- and stage-specific application strategies that maximize agronomic benefits while minimizing yield penalties.

## Data Availability

The raw data supporting the conclusions of this article will be made available by the authors, without undue reservation.
